# Survival rates of patients with metastatic malignant melanoma

**Published:** 2014

**Authors:** A Sandru, S Voinea, E Panaitescu, A Blidaru

**Affiliations:** * Department of Surgical Oncology, “Carol Davila” University of Medicine and Pharmacy; “Alexandru Trestioreanu” Oncologic Institute, Bucharest; ** Department of Medical Informatics and Biostatistics, “Carol Davila” University of Medicine and Pharmacy, Bucharest

**Keywords:** malignant melanoma, metastasis distribution, survival rates

## Abstract

**Rationale:** Malignant melanoma (MM) is the cutaneous neoplasia with the greatest mortality rates and one of the malignancies with the highest potential of dissemination. The prognosis of patients with metastatic MM is grim, with a 5-years survival rate between 5-19%, and is dictated by the location and the number of metastases.

**Objective:** We aimed to estimate the survival of patients with metastatic MM from our study and find out if the metastasis’ location influences survival.

**Methods and results:** Between 2008 and 2013, 155 patients with cutaneous MM were diagnosed in our clinic. All the patients were staged according to 2009 AJCC staging system. The median follow-up period was of 24 months. Survival was calculated by using the Kaplan-Meier method with a confidence level of 95%. 40.5% of the patients developed metastases in different organs, especially the brain. 80.6% of those with metastases died during the study. The median overall survival, estimated for the entire group of patients who developed metastases, was of 5.3 months.

**Discussion:** The influence of metastases distribution on the overall survival was examined and it was noticed that there were statistically significant differences between the risks of death of various groups of patients, depending on metastasis topography. Thus, the death probability of a patient with brain metastases is twice that of a patient with digestive metastasis, about 7 times higher than that of a patient with lung metastasis (p = 0.0004) and 12 times higher than the death risk of a patient with extra-regional lymph nodes or subcutaneous metastasis (p = 0.0000).

## Introduction

Cutaneous malignant melanoma (MM) represents a public health matter in many countries because its incidence has increased steadily over the past 30 years (Australia, New Zealand, South Africa, and the southern United States having the highest rates) and statistics estimate the doubling of incidence at every 10-20 years [**1**,**2**].

The incidence growth rate varies across different continents: in European population, MM incidence increases annually by 3-5% [**1**], and in United States population, by 5-7% [**3**].

To capture the magnitude of this rise, it is worth mentioning that while in 1935 the likelihood of a Caucasian individual to develop a MM was 1 in 1500, in 2011, this probability climbed to 1 in 50 [**3**].

However, the severity of the problem is not only an alarming increase of incidence but also the unexpected evolution of the disease and the inefficiency of the current systemic treatments.

MM has a great propensity for dissemination: after primary tumor excision, about 30% of the patients developed metastasis in various organs [**4**]. Except for stage 0 patients, in which surgery ensured healing and postoperative surveillance, it was only required to catch the event of a new MM occurrence (a risk of 3-5%), all the other patients with invasive MM might develop metastases, the greater the percentage, the more advanced the stage at diagnosis. The worry lies however in the fact that 5-15% of the patients with thin MM (tumor thickness < 1mm) develop metastases [**5**,**6**].

Recent data support the hypothesis that MM has a simultaneous lymphatic and hematogenous spread, with the potential to metastasize in any organ, but in different percentages. In various statistics, the frequency of metastases diagnosis in certain tissues varies according to the following ranges: skin ~ 10-60%, lung ~ 10-40%, extra-regional lymph nodes ~ 5-35%, subcutaneous tissue ~ 5-35%, CNS ~ 2 - 20%, liver ~ 14-20%, bone ~ 4-17%, adrenal glands ~ 1-11%, gastrointestinal tract ~ 1-8%, pleura ~ 5%, pancreas ~ 3%, heart, kidney, thyroid, uterus <1 % [**7**].

According to 2009 TNM (Tumor, Node, Metastasis) staging system, not all the metastases have the same prognosis. Balch et al. considered that stage IV patients` survival is directly influenced by the location of tumor dissemination and LDH serum concentration, delineating three categories of metastases according to the risk of death [**8**]:

• Skin, subcutaneous tissue and extra-regional lymph nodes metastasis (M1a), which were completely surgically resected, allowed a significantly higher OS than all the other locations, approximately 23% of the patients being alive at 5 years [**9**].

• Lung metastases (M1b) had a better prognosis than the rest of the visceral localization with a 5-year OS of 17% [**9**].

• Visceral metastases (M1c), except for lung, especially brain and liver, evolved in most of the cases to exitus, with a 5-year OS of less than 10% and a median survival (MS) below 1 year, despite modern treatments applied [**10**].

We tried to assess the factors that influenced the survival of patients with metastases treated in our clinic, and estimated the risk of death, median and overall survival.

## Material and method

155 patients with cutaneous MM were investigated between 2008 and 2013. The classification of patients in one of the five clinical stages was done according to the 2009 TNM staging version developed by AJCC (American Joint Commission on Cancer) and approved by the UICC (Union for International Cancer Control) [**8**].

The diagnosis of MM was established by excisional biopsy of the primary tumor in 153 patients, by inguinal lymphadenectomy in a patient with achromic melanoma of the hallux nail bed, and by excisional biopsy of a subcutaneous tumor (either an in transit metastasis – stage III disease, or a subcutaneous metastasis – stage IV disease) in a patient with MM of unknown primary site. At diagnosis: 2 patients had in situ tumors, 31 were enrolled in stage I, 72 in stage II, 47 in stage III and 3 in stage IV.

After the appropriate treatment according to each clinical stage, patients were submitted to periodic controls, at every 6 months, which consisted in a complete physical exam, abdominal and regional lymphatic basin ultrasound and chest radiography. If these routine tests have raised suspicion of metastasis, then the investigations were supplemented with computed tomography and, in a few cases, with positron emission tomography.

The patient’s survival was calculated by using the Kaplan-Meier method with a confidence interval (CI) of 95%. Log-rank test (Mantel-Cox) and Wilcoxon were used in univariate analysis. Prognostic value (p) of each covariable was determined by applying Cox model of proportional hazards (multivariate logistic regression). P values < 0.05 were considered statistically significant.

## Results

The median follow-up interval was of 24 months. Throughout the monitoring period, 14 local and regional relapses, 62 distant relapses, 58 deaths due to MM (37.4%) and another 2 deaths due to therapeutic complications (massive pulmonary embolism the next day after inguinal lymphadenectomy and acute myocardial infarction during high-dose interferon therapy), were recorded. At the end of the study, only half of the followed patients, (50.9%), were alive with no evidence of the disease, although two thirds of the enrolled patients were stage I and II at the initial diagnosis and therefore considered to have a good prognosis.

The overall survival rate for the whole lot was calculated from the date of MM diagnosis, that is histopathological analysis of a tumor specimen, until the end of the study or death registration date. For the disease free survival (DFS) the endpoint was the first occurrence of relapse (local, regional or distant). The five year OS for the entire group was 47% and five year DFS was 34%. The mean survival time was 69.7 months (95%CI = 6.7 – 56.6 month) and the median overall survival (MOS) was 53.4 months (95%CI =34.4 – 72.3 months). Distant disease progression was noticed in 40.5% of the cases (62 patients) and, until the end of the follow-up period, 80.6% of these patients were deceased.

For stage IV patients, all the survival indicators were calculated from the moment of metastasis discovery to the end of study or the patients’ death. The median overall survival, estimated for the group of patients who developed metastases, was of only 5.3 months (95%CI = 4.3 -6.3 months) and the mean survival was of 9.2 months (95%CI = 6.8 – 11.6 months).

**Fig. 1:**
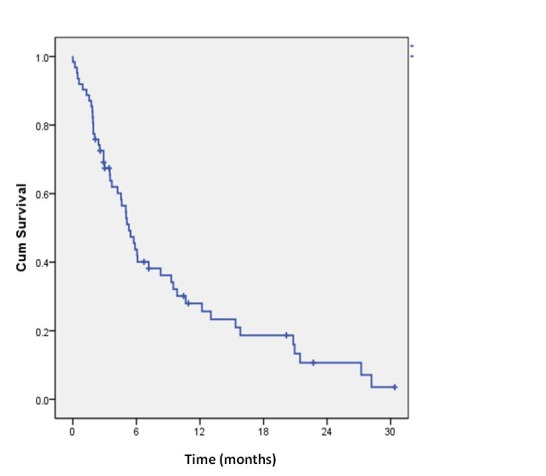
Kaplan-Meier overall survival for all stage IV patients: + censored patients

As it can be seen in **Fig. 1**, the one year OS of stage IV patients was 28% and the two-year OS was 10.7%.

The initial location of metastases in our patients was as it follows brain – 43.5% (27 patients), extra-regional lymph nodes and subcutaneous tissue - 27.4% (17 patients), lung - 14.5% [**9**], digestive tract - 12.9% [**8**], ovary – 1 patient. One third of stage IV patients (33.9%) had a simultaneous invasion of several organs, some of them being quite unusual: ovary, breast, great omentum. This multiple site dissemination had negatively influenced the survival.

We have found that MM patients with central nervous system (CNS) metastases, an M1c category, had the smallest median OS, of only 2.5 months (95%CI = 1.5 – 3.6 months). They were closely followed by patients with metastases in the liver and digestive tract, also an M1c category, with a median OS of 5.5 months (95%CI = 3.3 – 7.6 months), and those with lung metastases (M1b) - 13 months (95%CI = 0.0 – 28.9 months). The median OS of patients with subcutaneous and lymph nodes metastasis (M1a) was, as in all the other studies, the largest, 20.8 months (95%CI = 6.4 – 35.2 months).

**Fig. 2:**
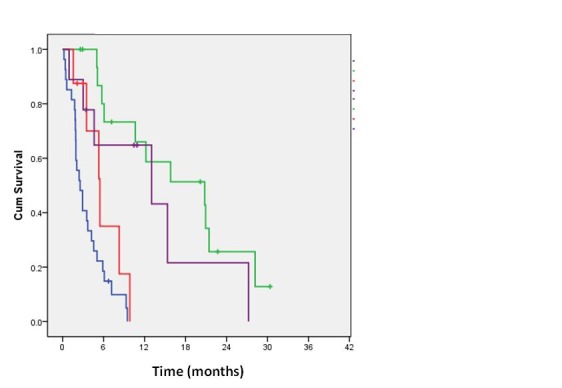
Kaplan Meier overall survival for stage IV MM patients depending on the first metastatic site Blue line – brain metastasis; red line – digestive metastasis; purple line – lung metastasis; green line - subcutaneous and extra-regional lymph nodes metastasis

The overall survival of stage IV patients depended on the site and the number of metastases. By using Cox regression, the impact of metastasis distribution on OS was analyzed and statistically significant differences between the death risks of various groups of patients, depending on metastases topography were found. Brain, lung, extra-regional lymphatic and digestive (both liver and gastrointestinal tract) metastatic development were chosen as independent variables.

Although the death probability of a patient with brain metastasis in the study was almost twice of a patient with digestive metastasis, hazard ratio (HR) being of 1.98, the difference was not statistically significant (p = 0.1333). However, for all the other metastatic sites taken into account, the risk of death from brain metastasis was significantly greater (p < 0.05).

Thus, a MM patient with brain metastasis had a risk of dying from his disease about 7 fold higher (HR = 6.8719) than that of a patient with lung nodules (p = 0.0004) and 12 times greater (HR = 12) than that of a patient with lymphatic or subcutaneous metastasis (p = 0.0000).

**Table 1:** Overall survival for stage IV patients according to first metastatic site

**Time**	**BRA**	**DYG**	**PUL**	**LYM+SC**
3 months	40.7%	87.5%	77.8%	100%
6 months	18.5%	35.0%	64.8%	80%
9 months	9.9%	17.5%	64.8%	73.3%
1 year	0%	0%	64.8%	66.0%
2 years	0%	0%	21.6%	25.7%
*BRA – brain metastasis; DYG – liver and gastrointestinal tract metastasis; PUL – lung metastasis; LYM+SC - extra-regional lymph nodes and subcutaneous metastasis*				

It was found that the presence of digestive metastasis, whatever segments were concerned (liver or gastrointestinal tract), significantly reduced OS. Their occurrence increased 3-fold the risk of death (HR = 3.458) compared with lung metastases (p = 0.0521) and 6 times (HR = 6.0419) versus lymphatic metastases (p = 0.0033).

Only patients with extra regional lymphatic and lung metastatic spread in the study were still alive after 2 years from the diagnosis of metastasis. The 2 years OS was of 21.6% for patients with lung metastasis and of 25.7% for those with skin, subcutaneous tissue and extra-regional lymph nodes metastasis.

What should also be mentioned is that the relatively small number of patients with particular metastasis location could influence all the statistical results.

## Discussions

Due to the public awareness of the risk of lentigo maligna transformation and access to screening, it was estimated that worldwide, the proportion of patients who presented to the doctor in stage IV decreased, reached no more than 8% [**10**]. Only 3 patients in the study already had metastases at the first consultation. Although most of the subjects diagnosed with stage I-III MM could be cured by surgery alone, almost a third of them had a disease progresses, and the risk of developing metastasis persisted throughout life [**11**].

Metastatic MM had a dismal prognosis with a high mortality rate. The 5-years overall survival (OS) ranged between 5-19% depending on the location and number of metastases and associated systemic changes (survival decreased if LDH values were elevated).

As it has been mentioned before in the study, the median overall survival for patients with metastases was of 5.3 months, smaller than that described in large series of patients, in average 7-8 months [**10**,**12**]. This result was not surprising, considering that about half of our patients (45.2%) also developed brain metastases, which portended the worst prognosis. The relatively high percentage of CNS tumors was likely responsible for the fact that the estimated 5-year OS for stage IV patients was 0% in our study, while in the other clinical trials the darkest predictions estimated a 5-year OS of about 5% [**12**].

MM is the third leading cause of brain metastases after lung and breast cancer. From all the adult malignancies, MM has the highest propensity to disseminate to the CNS. The information collected from the autopsy records revealed that 75% of the patients who died of MM, also had brain metastases [**13**].

The current treatment of brain metastasis is not satisfactory anywhere in the world. The median survival of these patients, observed in cohorts of hundreds of subjects, is generally less than 1 year, with a death probability of 95% in the first five years after diagnosis [**13**]. The 1 year OS of patients with brain dissemination was of 0% in our study, a rate lower than that of all the other metastatic sites.

Based on the review of thousands of files, literature data claimed that almost always (86%) dissemination initially occurs in just one organ [**10**]. And the organ first involved is the lung in 40% of the cases [**11**].

Only 6.5% of the patients in our lot had solitary lung metastases. In the other cases, lung nodules were associated with other metastatic sites, mainly lymph nodes and brain. Studying a cohort of 13 565 MM patients, Petersen et al. calculated that the risk of developing lung metastases increased over time, being of 13% at 5 years, 17% at 10 years and 23% at 20 years after the initial diagnosis of primary tumor [**11**]. It is somehow a surprising finding, given the fact that older information considered that the maximum risk of local and distant recurrence was in the first 5 years after diagnosis.

Although it may seem a paradox that a disseminated disease with a targeted treatment addressed strictly to an organ was approached, the best results in the treatment of single metastases was surgery, later followed by chemotherapy [**14**]. The only patients with lung metastasis, still alive in our study, were the two who underwent atypical pulmonary resection.

Most data showed 5-years OS for patients with lung metastasis of 29% in the most favorable cases, in which surgery was possible [**15**]. The 2-years OS of patients with lung metastases in the study was 21.6% and the estimated 5-years OS was 0%.

It seems that MM has a tendency, less common to other malignancies, to metastasize to the digestive system and especially to the small intestine mucosa, without a known pathogenic substrate of this behavior [**16**]. Sanki et al. claimed that more than 25% of the deceased MM patients showed metastases in at least one segment of the digestive tract at autopsy [**16**].

In most bibliographic sources, the survival indicators for patients with metastases in the digestive area were presented separately for liver and gastrointestinal tract, because the latter could require an emergency intervention (for obstruction or bleeding) and then a rigorous selection of patients was no longer possible.

Eight patients in the study developed digestive metastasis first: 4 of them only had liver tumors, other 3 patients presented with severe anemia consecutively to repeated melena and another one with subocclusive syndrome. These last 4 patients were subjected to surgery. During the exploratory surgery, multiple tumors were found distributed on both the small intestine and colon, some of them ulcerated with active bleeding source, other vegetant, with an almost complete obstruction of the lumen.

The location and number of tumors found made it impossible to target a radical intervention, and operations were limited to removing the obstacle or source of bleeding, by minimum intestinal resections. The outcome of these patients was not significantly influenced, because relapses were the rule and they all died within a few months.

However, there are authors who believe that palliative resections are justified in acute or subacute syndromes, primarily because they improve the quality of life, and in some cases may extend survival [**15**,**16**]. During 25 years (1981-2005), 142 surgeries for digestive metastases were practiced in the Sydney Melanoma Unit. Of these, 53 were palliative digestive resections, which were followed by a 34% 1-year OS and 19% 2-years OS, while the MS was 7.7 months [**16**].

The 4 patients in the study who developed liver metastases first had multiple tumors disseminated in both hepatic lobes. In all cases, polychemotherapy was chosen.

None of the patients in the study, whose first metastatic site was gastrointestinal, was alive one year after the diagnosis of metastasis.

## Conclusion

Although there are multiple controversies regarding all aspects of MM, there is a statement which almost everyone agreed with: MM is one of the most aggressive malignancies with an unpredictable evolution, a worrying mortality and a treatment with questionable results in advanced stages.

In this context, we believe that the survival of these patients can be improved only through a joint effort involving an early detection, complete staging process, properly treatment and a careful follow-up to detect the onset of any metastases. Also, in stage IV, an aggressive treatment addressed to carefully selected patients can provide an increase both in life quality and survival time.

**Disclosures:** authors declare no potential conflict of interests.

**Sources of Funding**

This paper was co-financed from the European Social Fund, through the Sectorial Operational Programme Human Resources Development 2007-2013, project number POSDRU/159/1.5/S/138907 “Excellence in scientific interdisciplinary research, doctoral and postdoctoral, in the economic, social and medical fields –EXCELIS”, coordinator Bucharest University of Economic Studies
